# To Unveil the Molecular Mechanisms of Qi and Blood through Systems Biology-Based Investigation into Si-Jun-Zi-Tang and Si-Wu-Tang formulae

**DOI:** 10.1038/srep34328

**Published:** 2016-09-28

**Authors:** Jing Sun, Li Zhang, Yujun He, Kun Zhang, Liping Wu, Yongsheng Fan, Zhijun Xie

**Affiliations:** 1 The Second Affiliated Hospital of Zhejiang Chinese Medical University, Hangzhou, Zhejiang, China; 2 Hangzhou Traditional Chinese Medical Hospital, Hangzhou, Zhejiang, China; 3 Zhejiang Chinese Medical University, Hangzhou, Zhejiang, China

## Abstract

Traditional Chinese Medicine (TCM) is increasingly getting clinical application worldwide. But its theory like QI-Blood is still abstract. Actually, Qi deficiency and blood deficiency, which were treated by Si-Jun-Zi-Tang (SJZT) and Si-Wu-Tang (SWT) respectively, have characteristic clinical manifestations. Here, we analyzed targets of the ingredients in SJZT and SWT to unveil potential biologic mechanisms between Qi deficiency and blood deficiency through biomedical approaches. First, ingredients in SWT and SJZT were retrieved from TCMID database. The genes targeted by these ingredients were chosen from STITCH. After enrichment analysis by Gene Ontology (GO) and DAVID, enriched GO terms with p-value less than 0.01 were collected and interpreted through DAVID and KEGG. Then a visualized network was constructed with ClueGO. Finally, a total of 243 genes targeted by 195 ingredients of SWT formula and 209 genes targeted by 61 ingredients of SJZT were obtained. Six metabolism pathways and two environmental information processing pathways enriched by targets were correlated with 2 or more herbs in SWT and SJZT formula, respectively.

Traditional Chinese Medicine (TCM), being an effective treatment system, is increasingly getting clinical application worldwide. After more than 5000 years of clinical practice, nearly 100,000 classical and effective TCM formulae have been developed, but the effective mechanisms of most formulae remain unclear^1^. The abstract and subjective theories like Yin-Yang and QI-Blood theories are still the main obstacle for application of TCM worldwide. Actually, Qi deficiency and blood deficiency have characteristic clinical manifestations. The clinical manifestations should be based on objective pathological change at gene or protein level. Similar with western medicine, ingredients of Chinese medicine have their targets (proteins or genes, etc.), which is the key factor to bridge the gap between western medicine and TCM.

Since the TCM formulae are normally composed of several medicinal herbs, and each herb normally has many ingredients, and each ingredient has a lot of targets, a formula is a complex biologic active network. Fortunately, along with the rapid development of life science and computer science, a variety of computational tools and bioinformatic database have been developed to facilitate the analysis of a large number of genes associated with complex ingredients of TCM formulae^2^, which provide opportunities to predict potential pharmacological actions of TCM formulae and clarify complex molecular mechanisms of formulae and theories of TCM. Based on primary biomolecular databases, e.g. Kyoto Encyclopedia of Genes and Genomes (KEGG, http://www.kegg.jp)^3^, HPRD^4^, PDB^5^, TTD^6^, OMIM^7^, Drug-Bank^8^, STITCH^9^ and ChEMBL^10^, a lot of TCM-related databases have been developed, such as TCMID^11^, HIT^12^, TCM Database@Taiwan^13^, TCMGeneDIT^14^, TCM-ID^15^, TCMSP^16^ and CHMIS-C^17^. These TCM-related databases complement each other to provide information on complex interactions of TCM-active ingredient-gene-disease^2^. Among these TCM-related databases, TCMID (http://www.megabionet.org/tcmid/) contains 3,791 diseases, 47,000 prescriptions, 8,159 herbs, 6,828 drugs, 25,210 compounds and 17,521 related targets, which facilitates the study of interactions between formula, ingredient, gene and disease to uncover the molecular biological mechanisms of TCM. Meanwhile, there are several network analysis tools for biological functionality of TCM-related network analysis, such as Cytoscape^18^,^19^. More than 150 specialized plugins integrated in Cytoscape can be used to import and map existing interaction data cataloged in public databases^2^, such as ClueGO^20^, BioGrid Plugin^21^ and MiMI^22^. ClueGO integrates Gene Ontology (GO) terms as well as KEGG/BioCarta pathways to create functionally organized GO/pathway term network and analyze one or compare two lists of genes and comprehensively visualizes functionally grouped terms.

Increasing TCM researchers successfully analyzed pharmacological mechanism of TCM formulae by using TCM-related databases and data analysis tools^23^,^24^,^25^,^26^. In trying to unveil the different potential biologic mechanisms between Qi deficiency and blood deficiency through biomedical approaches, we chose SJZT and SWT, which have been used in China and other Asian countries for about 1,000 years to effectively rectify Qi deficiency and blood deficiency, respectively. SJZT consisting of Panax ginseng, Atractylodes macrocephala, Poria cocos and Radix Glycyrrhizae Preparata, is the basic TCM prescription of tonifying Qi. SWT consisting of Rehmannia glutinosa, Angelica Sinensis, Ligusticum chuanxiong and Paeonia albiflora, is the TCM classical prescription of nourishing blood. SJZT and SWT were both recorded in < Taiping and the agent of the bureau party > , which was the first national pharmacopoeia and was published in twelfth Century in the Song Dynasty of China. The research flow chart was shown in Fig. 1.

## Results

### Ingredients and targets of SWT and SJZT

**Figure 2:**
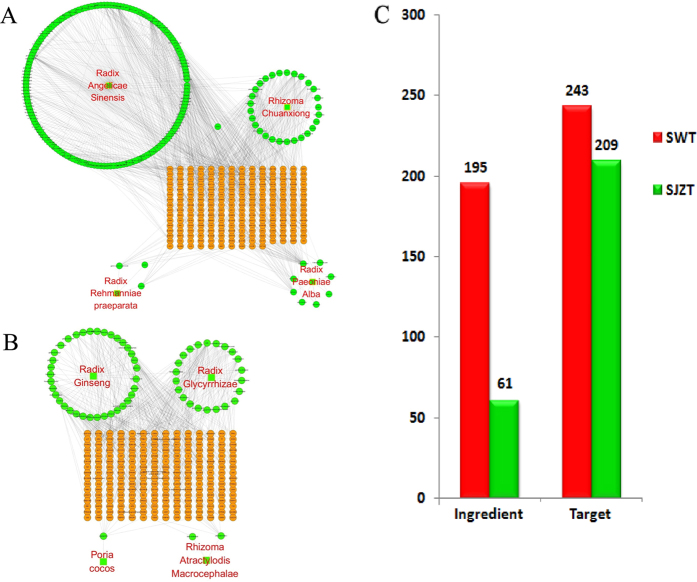
Counts of ingredients and genes targeted by SWT and SJZT formulae. (**A**) The herb-ingredient-target network of SWT; (**B**) The herb-ingredient- target network of SJZT; (**C**) comparison of count of ingredients and targets of SWT and SJZT; Red, SWT, Green, SJZT. In (**A,B**), the square nodes represent herbs, green round nodes represent ingredients and yellow round nodes represent targets (genes).

As shown in Retrieving from TCMID, we obtained 152 genes targeted by 162 ingredients of Radix Angelicae Sinensis, 107 genes targeted by 28 ingredients of Ligusticum chuanxiong, 21 genes targeted by three ingredients of Rehmannia glutinosa, 42 genes targeted by eight ingredients of Paeonia albiflora (Fig. 2A), and 156 genes targeted by 37 ingredients of Panax ginseng, nine genes targeted by two ingredients of Atractylodes macrocephala, nine genes targeted by one ingredients of Poria cocos and 62 genes targeted by 22 ingredients of Radix Glycyrrhizae Preparata (Fig. 2B). After screened according to STITCH combined-score more than 0.7, a total of 243 genes targeted by 195 ingredients of SWT formula and 209 genes targeted by 61 ingredients of SJZT (Fig. 2C) were obtained. The entire list of 452 genes targeted by 256 ingredients of SJZT and SWT can be found as Supplementary Table S1.

### Comparison of pathways between SWT and SJZT formulae

**Figure 3:**
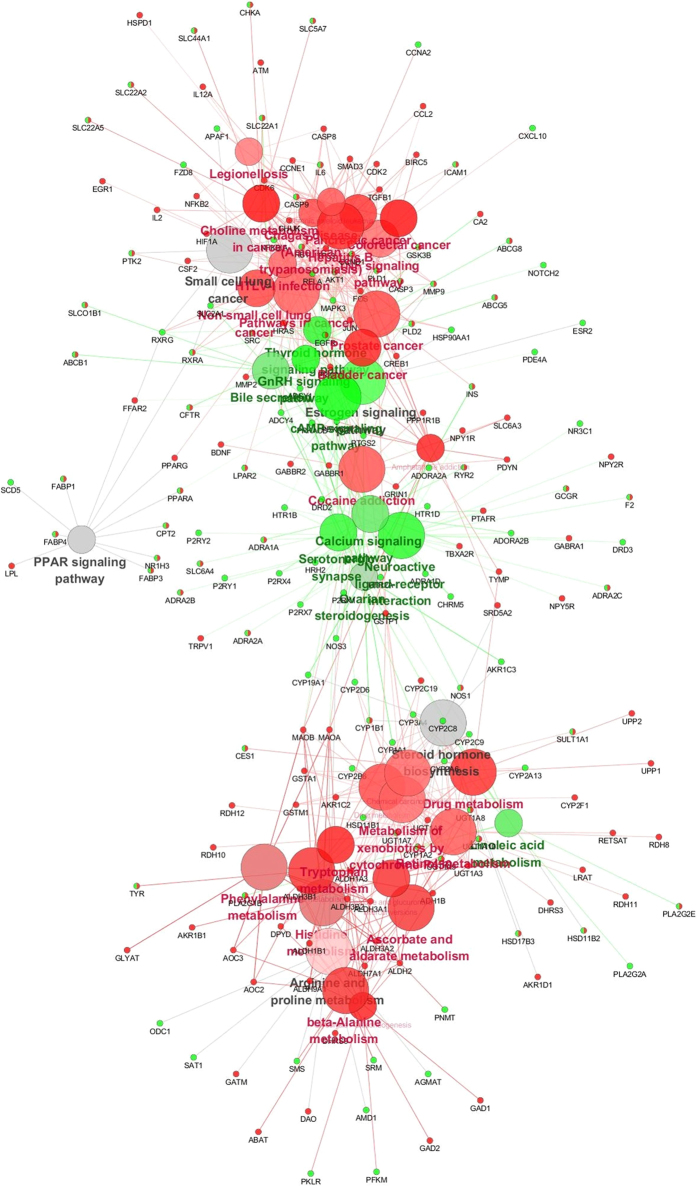
Network of pathways enriched by genes targeted by SWT and SJZT. ClueGO KEGG analysis of predicted targets of SWT and SJZT. GO terms are represented as nodes, and the node color depth represents different proportions of genes/proteins of SWT and SJZT in each grouped term, the node size represents the term enrichment significance. Edge represents the relationship between terms. Red nodes represent terms of SWT; green nodes represent terms (pathways) of SJZT; grey nodes represent common terms of the two formulae. Functionally related terms partially overlap.

Interestingly, there were obviously three main grouped clusters independent from each other in the network (Fig. 3), two clusters (amino acid and carbohydrate metabolism and disease associated pathways) belonged to SWT and the other cluster (pathways mainly connected with signal transduction, endocrine hormone secretion and lipid metabolism) belonged to SJZT.

After analysis of pathway associated genes, we found that there were six metabolism pathways, including beta-Alanine metabolism, histidine metabolism, phenylalanine metabolism, tyrosine metabolism, ascorbate and aldarate metabolism, glycolysis/gluconeogenesis pathways, were correlated with 2 or more herbs in SWT formula and two environmental information processing pathways, calcium signaling pathway and neuroactive ligand-receptor interaction pathway were correlated with 2 or more herbs in SJZT formula (Table 1).

We also found that there were many disease associated pathways were enriched. According the differentiating criterion that if more than 66% of the genes targeted by ingredients of one formula associate with a term (pathway), the term (pathway) is considered specific for the formula, the disease associated pathways were differentiated into two categories, i.e. common pathway and different pathway. The common pathway, including viral carcinogenesis, thyroid cancer, chemical carcinogenesis, legionellosis and small cell lung cancer pathways are unspecific for SWT or SJZT. The different pathway were all specific for SWT, including amphetamine addiction, colorectal cancer, choline metabolism in cancer, bladder cancer, hepatitis B, pancreatic cancer, non-small cell lung cancer, prostate cancer, chagas disease (American trypanosomiasis), cocaine addiction, chronic myeloid leukemia, HTLV-I infection, p53 signaling pathways and pathways in cancer (Table 2).

### Non-disease associated pathways between SWT and SJZT

As mentioned above, there were many cancer and other diseases associated pathways in the network (Fig. 2), which might interfere with the correct judgment of the main characteristics of Qi deficiency and blood deficiency. So we collected the genes in non-disease pathways and deleted the same targets of SWT and SJZT for further analysis. Finally, we constructed a non-disease associated network to compare the main difference in pathways between SWT and SJZT or Qi deficiency and blood deficiency.

**Figure 4:**
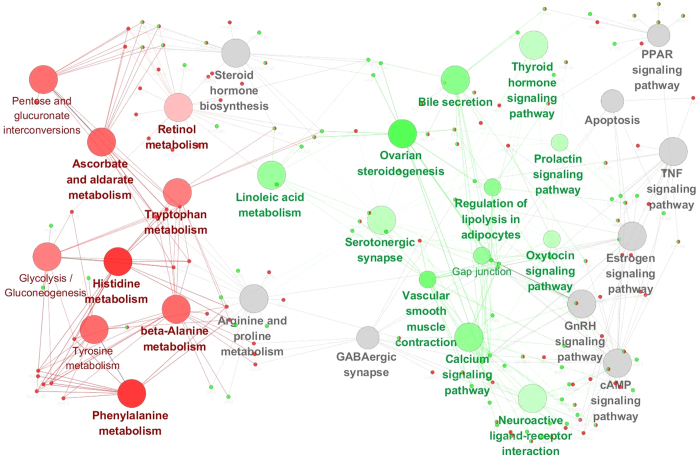
Network of non-disease pathways enriched by SWT and SJZT. ClueGO KEGG analysis of non-disease pathways of SWT and SJZT.GO terms are represented as nodes, and the node color depth represents different proportions of genes/proteins of SWT and SJZT in each grouped term, the node size represents the term enrichment significance. Edge represents the relationship between terms. Red nodes represent terms of SWT; green nodes represent terms (pathways) of SJZT; grey nodes represent common terms of the two formulae.

In the non-disease associated network, pathways enriched with genes targeted by ingredients of SWT were mainly involved in material metabolism (i.e. amino acid metabolism, carbohydrate metabolism and metabolism of cofactors and vitamins), including beta-Alanine metabolism, histidine metabolism, phenylalanine metabolism, tyrosine metabolism, tryptophan metabolism, ascorbate and aldarate metabolism, pentose and glucuronate interconversions, glycolysis/gluconeogenesis, retinol metabolism pathways (Fig. 4 and Table 3). Retinol metabolism pathway is related with amino acid metabolism, carbohydrate metabolism.

While pathways enriched by genes targeted by ingredients of SJZT mainly belonged to organismal system function, environmental information processing, metabolism and cellular processes pathways. The organismal system function pathways included thyroid hormone signaling pathway, ovarian steroidogenesis, prolactin signaling pathway and oxytocin signaling pathway (endocrine system), bile secretion pathway (digestive system), vascular smooth muscle contraction pathway (circulatory system) and serotonergic synapse (nervous system). The environmental information processing pathways included neuroactive ligand-receptor interaction pathway (signaling molecules and interaction) and calcium signaling pathway (Signal transduction). The metabolism pathways of SJZT were different to SWT, which mainly belong to lipid metabolism including linoleic acid metabolism and regulation of lipolysis in adipocytes pathways. The cellular processes pathway included Gap junction (cellular community), (Fig. 4 and Table 3).

In the network of non-disease associated pathways enriched by SWT and SJZT, there also were several pathways with *p* value less than 0.01 unspecific for SWT or SJZT. We considered them as the common pathways of SWT and SJZT. These pathways also belonged to organismal systems, metabolism, environmental information processing and cellular processes, including estrogen signaling pathway (endocrine system), GnRH signaling pathway (endocrine system), PPAR signaling pathway (endocrine system), GABAergic synapse (nervous system), Arginine and proline metabolism (amino acid metabolism), Steroid hormone biosynthesis (lipid metabolism), Metabolism of xenobiotics by cytochrome P450 (Xenobiotics biodegradation and metabolism), cAMP signaling pathway (signal transduction), TNF signaling pathway (signal transduction), Apoptosis (Cell growth and death), (Table 4).

### Target prediction

In order to clarify the difference between SWT and SJZT at gene level, we also screened different genes from differentiated pathways between SWT and SJZT (Table 5).

## Discussion

Combination therapy is the major feature of TCM, which is increasingly recognized by modern western medicine, such as cocktail therapy for HIV^27^ and the opinion shifting from targeting a single disease-causing molecule to the pursuit of combination therapies that comprise more than one active ingredient^28^. According to the symptoms of patients, different kinds of Chinese medicines are combined to form formulae to improve clinical efficacy^1^. such as SWT and SJZT have been used to rectify blood deficiency and Qi deficiency respectively for about 1,000 years.

Blood deficiency normally manifests anaemia, vertigo, heart palpitations and menstrual discomfort. The SWT formula has effects on stimulating hematopoiesis in bone marrow, anti-coagulant, vasodilatation and sedative^29^,^30^, so it can be used to treat anemia^31^, bone formation^32^ dysmenorrhea^33^,^34^ and other estrogen-related diseases^35^,^36^. Qi deficiency normally manifests lack of strength, body function decline and decreased disease resistance, and so on. The SZJT formula has effects on regulating granulocyte macrophage colony-stimulating factor secretion^37^, enhancing phagocytosis of macrophages^38^, recovering cAMP signal pathway^39^ and recovery of intestinal microflora^40^. However, the mechanisms of the pharmacological action of SWT and SJZT have not yet been clarified.

Applying syndrome differentiation through formula effect assessment, we could predict the pathways associated with blood deficiency and Qi deficiency syndromes according to the pathways enriched by SWT and SJZT, and then indirectly predict the molecular mechanism of blood and Qi.

Interestingly, when using targets by ingredients of SWT and SJZT to enrich and construct the Network (Fig. 2), there were obviously three main clusters independent from each other, and two clusters (amino acid and carbohydrate metabolism and disease associated pathways) belonged to SWT, and the other cluster (pathways mainly connected with signal transduction, endocrine hormone secretion and lipid metabolism) belonged to SJZT. Because cancer and other diseases associated pathways would interfere with the correct judgment of the main characteristics of Qi deficiency and blood deficiency, we further constructed a non-disease associated pathways visualized network to compare the main difference in pathways between SWT and SJZT or Qi deficiency and blood deficiency.

Except for disease associated pathways, SWT significantly influence amino acid and carbohydrate metabolism (beta-Alanine metabolism, histidine metabolism, phenylalanine metabolism, tyrosine metabolism, tryptophan metabolism, ascorbate and aldarate metabolism, glycolysis/gluconeogenesis, pentose and glucuronate interconversions and retinol metabolism pathways), which are closely related with nutrient substance. Abnormal glycolysis/gluconeogenesis was also found by another study about urine metabonomic in blood-deficient mouse model^41^. According to the theory of TCM, the blood, one kind of nutrient substance belonging to the category of Yin, has the function of nourishing general organs. The results of this study implicate the function of blood is closely related with amino acid and carbohydrate metabolism, which is consistent with the blood theory of TCM.

Different from blood of TCM, Qi has functions of promoting substance metabolism and energy conversion, stimulating activity of organs, keeping blood circulating in vasculature, promoting human growth and development, maintaining normal temperature of human body, strengthening the ability of anti-infection, maintaining normal development of fetus in uterus and controlling the secretion and excretion of bile, sweat, urine, saliva, gastric and intestinal digestive juice.

This study found that SJZT could influence several syntrophic pathways. Among them, bile secretion, linoleic acid metabolism, regulation of lipolysis in adipocytes pathways can affect bile secretion and lipid metabolism and then influencing lipid hormones synthesis; neuroactive ligand-receptor interaction, serotonergic synapse, gap junction pathway can influence the function of neuroendocrine system; thyroid hormone signaling pathway, ovarian steroidogenesis, prolactin signaling pathway, oxytocin signaling pathway can regulate corrective hormones secretion; vascular smooth muscle contraction can regulate the blood flow and pressure and keep blood circulating in vasculature; while calcium signaling pathway involve in several pathways above. Tian, R. *et al.*^42^ also found out that SJZT may achieve the therapeutic effect in Qi deficiency syndrome by increasing the calmodulin expression in hippocampus tissues and Qi deficiency syndrome may be related to the low expression of calmodulin in hippocampus tissues. And Duan, Y. Q. *et al.*^43^ found out that SJZT can rectify qi deficiency syndrome by regulating gastrointestinal hormones (GAS and MOT) secretion and raising the expressions of key factors of Ca2+ /CaM signaling pathways in skeletal muscle tissue. As we know, the activity of calmodulin only in combination with Ca^2+^ to have activity. And, the hormone can influence the activity of calmodulin by regulating the concentration of intracellular Ca^2+^. Thyroid hormone has function of promoting human growth and development, calorigenic effect on maintaining normal temperature of body and function of stimulating substance metabolism and energy conversion, which can stimulate activity of organs. Ovarian steroidogenesis, prolactin signaling pathway, oxytocin signaling pathway can help to maintain normal development of fetus in uterus. All the functions of pathways enriched by SJZT are consistent with the actions of Qi of TCM.

In order to clarify the difference between SWT and SJZT at gene level, we also screened different genes from differentiated pathways between SWT and SJZT (Table 5). These genes can used as molecular targets and help us to study the different molecular mechanism of SWT and SJZT in next clinical research, which may also help to clarify the molecular mechanism of Qi deficiency and blood deficiency.

In TCM theories, blood and Qi also have close relationship, i.e. Qi can promote the formation and circulation of blood. Meanwhile blood can nourish Qi. In this study, we also found that there were many common pathways between SWT and SJZT, which reflects the close relationship between Qi and blood.

As shown in the network (Fig. 4), there were also close relationship between these common pathways. GABA are the principal inhibitory neurotransmitters of the entire central nervous system including the hypothalamus^44^, so GABAergic synapse play key regulatory roles in the control of GnRH signaling pathway^45^. GnRH binds to its receptors on the gonadotropes and stimulates the release of the gonadotropins, luteinizing hormone (LH)^46^ and follicle-stimulating hormone (FSH), then stimulate estrogen signaling pathway and steroid hormone biosynthesis to release steroid hormones (estrogens, progestins, and androgens in both females and males)^47^, and cAMP signaling pathway mediates these processes. All above pathways reflect the functional activities of the hypothalamic pituitary adrenal and gonadal axis. Estrogen and steroid hormones can promote hematopoiesis^48^, maintain normal menses and the development of fetus in uterus. Arginine is a key player in immune system^49^,^50^, it can induces growth hormone (GH) gene expression and activate NOS/NO to increase blood flow^51^, so regulating arginine and proline metabolism pathway is benefit for strengthening the ability of anti-infection, promoting human growth and development and increasing blood flow. PPAR signaling plays the important role in lipid metabolism. Cytochrome P450 is involved in metabolism of oxysterols, sex hormones and neurosteroids^52^, so metabolism of xenobiotics by cytochrome P450 is also benefit for lipid metabolism, sex hormones and neurosteroids secretion. Tumor necrosis factor (TNF), as a critical cytokine, can induce a wide range of intracellular signal pathways including apoptosis and cell survival as well as inflammation and immunity. Apoptosis is a genetically controlled mechanism of cell death involved in the regulation of tissue homeostasis. So the effects of SWT and SJZT on regulating the TNF signaling pathway and apoptosis pathway are helpful for strengthening immunity.

Above all, the common pathways are mainly associated with the effects of SWT and SJZT on strengthening the ability of anti-infection, promoting human growth and development and increasing blood flow and promoting hematopoiesis, maintaining normal development of fetus in uterus, rectifying dysmenorrhea and other estrogen-related diseases, which are consistent with the actions of blood and Qi of TCM.

In conclusion, SWT with the functions of influencing amino acid and carbohydrate metabolism is significant different from SJZT with the actions of influencing neuroendocrine system by affecting excitatory synapses (serotonergic synapse) to regulate thyroid hormone, ovarian steroidogenesis, prolactin and oxytocin secrction and can regulate corrective hormones secretion and promoting vascular smooth muscle contraction. The common effects of SWT and SJZT are regulating the functional activities of the hypothalamic pituitary adrenal and gonadal axis by affect inhibitory synapses (GABAergic synapse) to stimulate estrogen and steroid hormones secretion and strengthening the ability of anti-infection. All the differences and common pathways also reflect the characteristics of blood deficiency and Qi deficiency, and the molecular mechanism of blood and Qi of TCM.

## Methods

### Data collection

Ingredients of eight herbs from SWT (Angelicae sinensis, Ligusticum chuanxiong, Rehmannia glutinosa and Paeonia albiflora) and SJZT (Panax ginseng, Atractylodes macrocephala, Poria cocos and Radix Glycyrrhizae) were retrieved from TCMID database, 449 ingredients and 454 targets of SWT and 545 ingredients and 568 targets of SJZT were collected and organized. In this study, Only human genes with STITCH defined high confident (combined-score more than 0.7) were chosen^9^. All the targets were processed into consistent symbols by searching in HGNC (HUGO Gene Nomenclature Committee)^53^.

### Gene ontology and pathway enrichment analysis

454 and 568 genes targeted by ingredients of SWT and SJZT were performed Gene Ontology (GO) and pathway enrichment analysis with DAVID Bioinformatics Resources 6.7 (http://david.abcc.ncifcrf.gov/)^54^, respectively. Enriched GO terms (pathways) with *p*-value less than 0.01(corrected with Bonferroni step down) were collected and analyzed to interpret the biological meanings of these targeted genes datasets with comprehensive set of functional annotation tools of DAVID and KEGG.

### Network construction and comparison

Based on ClueGO (a Cytoscape plug-in)^20^, we tried to decipher functionally grouped gene ontology and pathway annotation networks. First, we used two clusters, including 454 genes of SWT and 568 genes of SJZT respectively, to construct a visual network to compare the different pathways between the two formulae. Because there were many disease associated pathways in the network, we collected the genes in the non-disease pathways according to the data of node attribute table produced by ClueGO for further analysis. Finally, we constructed a non-disease network to compare the difference between SWT and SJZT. The main parameters of constructing network with ClueGO were as follows, marker list: Homosapiens; ontologies/pathways: KEGG-kegg-293 terms/pathways with 6961 avaliable unique genes; showing only pathways with p-value less than 0.01; GO term/pathway network connectivity (Kappa score) was 0.7; if no less than 66% of the genes targeted by ingredients of one formula associate with a term (pathway), the term (pathway) is considered specific for the formula; statistical option: enrichment/depletion (two-sided hypergeometric test) with Bonferroni step down *p*-value correction.

## Additional Information

**How to cite this article**: Sun, J. *et al.* To Unveil the Molecular Mechanisms of Qi and Blood through Systems Biology-Based Investigation into Si-Jun-Zi-Tang and Si-Wu-Tang formulae. *Sci. Rep.* **6**, 34328; doi: 10.1038/srep34328 (2016).

## Supplementary Material

Supplementary Table S1

## Figures and Tables

**Figure 1:**
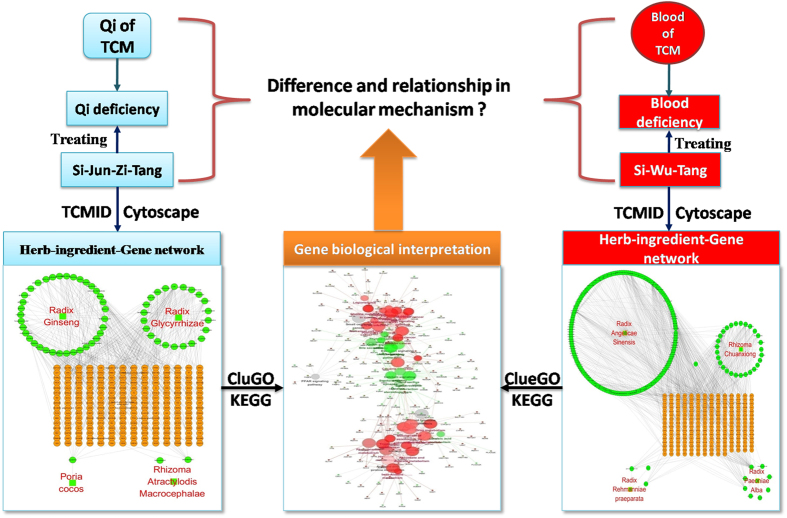
Research flow chart. We analyzed targets of the ingredients in Si-Jun-Zi-Tang (SJZT) and Si-Wu-Tang (SWT) to unveil the difference and relationship in molecular biological mechanisms between Qi deficiency and blood deficiency through biomedical approaches. First, ingredients in SWT and SJZT were retrieved from TCMID database. Then a visualized network of KEGG pathways was constructed with ClueGO to unveil the difference and relationship between Qi and blood.

**Table 1:** Common pathways enriched by targets correlated withmore thanoneherbs.

Formula	Herbs	Term of pathway	Category of pathway
SWT	Rhizoma Chuanxiong, Radix Angelicaesinensis, Radix RehmanniaePraeparata	beta-Alanine metabolism	Metabolism; Amino acidmetabolism
	Rhizoma Chuanxiong, Radix Angelicaesinensis, Radix RehmanniaePraeparata	Histidine metabolism	Metabolism;Amino acid metabolism
	Rhizoma Chuanxiong, Radix Angelicaesinensis	Phenylalanine metabolism	Metabolism; Amino acid metabolism
	Rhizoma Chuanxiong, Radix Angelicaesinensis	Tyrosine metabolism	Metabolism; Amino acid metabolism
	Rhizoma Chuanxiong, Radix RehmanniaePraeparata	Ascorbate and aldarate metabolism	Metabolism; Carbohydrate metabolism
	Rhizoma Chuanxiong, Radix RehmanniaePraeparata	Glycolysis/Gluconeogenesis	Metabolism; Carbohydrate metabolism
SJZT	Panax ginseng, Poriacocos	Calcium signaling pathway	Environmental Information Processing; Signal transduction
	Panax ginseng, Poriacocos, Radix GlycyrrhizaePreparata	Neuroactive ligand-receptor interaction	Environmental Information Processing; Signaling molecules and interaction

**Table 2:** Disease associated pathways enriched by targets of SWT and SJZT (*P* value < 0.01).

Category	GO Term (Pathway)	*P* value	Rate of targeted genes in pathway (%)	
Common pathway			SWT	SJZT
	Viral carcinogenesis	1.19E-03	64.25	49.42
	Thyroid cancer	2.91E-03	63.26	50.61
	Chemical carcinogenesis	8.67E-20	65.77	51.92
	Legionellosis	6.46E-04	62.84	53.86
	Small cell lung cancer	4.81E-07	61.67	61.67
	Amphetamine addiction	7.76E-05	83.33	16.67
	p53 signaling pathway	4.43E-03	80.79	35.91
	Colorectal cancer	3.27E-05	80.40	43.85
	Choline metabolism in cancer	5.20E-06	79.78	58.51
	Bladder cancer	3.74E-05	76.52	51.02
	Hepatitis B	1.01E-12	75.53	44.63
	Pancreatic cancer	7.90E-06	73.71	53.61
Different pathway	Non-small cell lung cancer	9.96E-06	71.64	57.31
	Prostate cancer	1.06E-09	71.35	52.32
	Chagas disease (American trypanosomiasis)	5.00E-05	70.09	38.23
	Cocaine addiction	1.66E-07	69.23	30.77
	Chronic myeloid leukemia	2.03E-04	68.82	45.88
	Pathways in cancer	2.44E-12	67.36	54.32
	HTLV-I infection	8.79E-07	66.33	48.88

**Table 3:** Difference of non-disease associated pathways of SWT and SJZT(*P* value < 0.01).

Formula	GO Term (Pathway)	Number of targeted genes	*P* value	Category of pathway
SWT	beta-Alanine metabolism	15	1.55E-16	Metabolism; Amino acid metabolism
	Histidine metabolism	11	2.24E-09	Metabolism; Amino acid metabolism
	Phenylalanine metabolism	9	5.93E-08	Metabolism; Amino acid metabolism
	Tyrosine metabolism	10	4.88E-07	Metabolism; Amino acid metabolism
	Tryptophan metabolism	10	2.94E-05	Metabolism; Amino acid metabolism
	Ascorbate and aldarate metabolism	11	1.93E-08	Metabolism; Carbohydrate metabolism
	Pentose and glucuronateinterconversions	10	9.88E-06	Metabolism; Carbohydrate metabolism
	Glycolysis / Gluconeogenesis	10	7.76E-05	Metabolism; Carbohydrate metabolism
	Retinol metabolism	16	3.96E-16	Metabolism; Metabolism of cofactors and vitamins
SJZT	Neuroactive ligand-receptor interaction	23	3.60E-10	Environmental Information Processing; Signaling molecules and interaction
	Calcium signaling pathway	18	1.46E-06	Environmental Information Processing; Signal transduction
	Gap junction	9	7.89E-03	Cellular Processes; Cellular commiunity
	Vascular smooth muscle contraction	13	7.35E-03	Organismal Systems; Circulatory system
	Bile secretion	13	2.31E-06	Organismal Systems; Digestive system
	Thyroid hormone signaling pathway	12	4.69E-05	Organismal Systems; Endocrine system
	Ovarian steroidogenesis	10	2.61E-04	Organismal Systems; Endocrine system
	Prolactin signaling pathway	8	7.27E-03	Organismal Systems; Endocrine system
	Oxytocin signaling pathway	11	8.65E-03	Organismal Systems; Endocrine system
	Serotonergic synapse	12	3.60E-06	Organismal Systems; Nervous system
	Linoleic acid metabolism	7	2.39E-04	Metabolism; Lipid metabolism
	Regulation of lipolysis in adipocytes	8	5.56E-03	Metabolism; Lipid metabolism

**Table 4:** Commonnon-disease associated pathways of SWT and SJZT (P value < 0.01).

GO Term (Pathway)	*P* value	Rate of targeted genes in pathway (%)		Category of pathway
		SWT	SJZT	
Estrogen signaling pathway	7.70E-09	54.37	59.31	Organismal Systems; Endocrine system
GnRH signaling pathway	3.61E-04	55.71	62.68	Organismal Systems; Endocrine system
PPAR signaling pathway	7.79E-04	67.88	67.88	Organismal Systems; Endocrine system
GABAergic synapse	1.46E-03	64.59	40.37	Organismal Systems; Nervous system
Arginine and proline metabolism	3.50E-12	57.66	46.13	Metabolism; Amino acid metabolism
Steroid hormone biosynthesis	3.48E-12	64.07	64.07	Metabolism; Lipid metabolism
Metabolism of xenobiotics by cytochrome P450	7.97E-20	65.08	50.62	Metabolism; Xenobiotics biodegradation and metabolism
cAMP signaling pathway	3.69E-10	54	60.75	Environmental Information Processing; Signal transduction
TNF signaling pathway	2.74E-06	64.7	53.92	Environmental Information Processing; Signal transduction
Apoptosis	1.03E-03	53.53	61.17	Cellular Processes; Cell growth and death

**Table 5:** Different targets in pathways enriched by SWT and SJZT (P value < 0.01).

Formula	Target (Genes/proteins)
SWT	*abat, adh1b, akr1b1, aldh1a3, aldh1b1, aldh2, aldh3a1, aldh3a2, aldh3b1, aldh3b2, aldh7a1, aldh9a1, aoc2, aoc3, dhrs3, dhrs9,dpyd, gad1, gad2, glyat, lrat, maoa, maob, rdh10, rdh11, rdh12, rdh8, retsat, tyr, ugt1a10, ugt1a3, ugt1a6,ugt1a7, ugt1a8, ugt1a9*
SJZT	*abcb1, abcg5, abcg8, adcy1, adcy4, adora2a, adora2b, adra1a, adra1d, adra2a, adra2b, adra2c, akr1c3, akt1, casp3, casp9, ccnd1, cftr, chrm5, cyp19a1, cyp1a1, cyp2c8, cyp2c9, cyp2d6, cyp3a4, drd2,drd3, egfr, esr2, f2, fabp4, gcgr, gsk3b, hrh2, htr1b, htr1d, htr2a, ins, lpar2, mapk3, nos1, nos3, notch2, nr3c1, p2rx1, p2rx4, p2ry1, p2ry2, pla2g1b, pla2g2a, pla2g2e, prkaca, prkacb, ptgs2, rela, rxra, rxrg, ryr2, slc22a1, slc2a1, slc6a4, slc01b1, src*
